# Development and characterization of microsatellite markers for the French endemic *Angelica heterocarpa* (Apiaceae) and congeneric sympatric species

**DOI:** 10.1186/s13104-021-05903-2

**Published:** 2022-01-10

**Authors:** Emmanuelle Revardel, Olivier Lepais

**Affiliations:** University of Bordeaux, INRAE, BIOGECO, 33615 Pessac, France

**Keywords:** Plants, Conservation, Population genetics, Genetic diversity, Nuclear SSR, Hybridization, Estuary riverbanks, *Angelica sylvestris*, *Angelica razulii*, *Angelica archangelica*

## Abstract

**Objective:**

*Angelica heterocarpa* (Apiaceae) is a wild endemic French species with special conservation interest in the European Union. It belongs to *Angelica* complex genus which is widespread throughout the north temperate zone, and is sympatric with other congeneric species*.* The objective of this work is to develop and characterize microsatellite markers as a new tool for understanding the ecology and evolution of *Angelica* species complex.

**Results:**

We identified simple sequence repeat (SSR) regions in a microsatellite‐enriched library from *A. heterocarpa* and *A. sylvestris*. All 16 selected SSR regions were found to amplify in these species and were highly polymorphic. Marker transferability was validated in *A. razulii* and *A. archangelica.* These markers will help us to better understand the evolutionary dynamic between rare endemics and widespread sister species, and be useful for conservation of the endemic species. Moreover, they can provide new tools for studying the numerous traditional medicinal herbs of the *Angelica* genus.

## Introduction

*Angelica* L. is a large complex genus comprising approximately 110 species confined in the northern hemisphere, with the majority in Eurasia [1]. In this paper, four French native congeneric *Angelica* species are considered. *Angelica heterocarpa* Lloyd is endemic to southwestern French estuary banks. It is protected at national level in France and is listed as priority species in the Habitats Directive of European Union [2]. *Angelica sylvestris* L. is common in open and forest habitats and is widely distributed among Europe and Asia. Despite different ecological niches, *A. heterocarpa* and *A. sylvestris* can live in neighboring riverbank locations. The observation of morphological intermediates questions the taxonomic relationship and potential for hybridization between these two species. Two other species are present in southwestern France: *Angelica razulii* Gouan, a Pyrenean endemic that shares hydrographic zones with *A. heterocarpa* and *A. sylvestris*, and *Angelica archangelica* L. that occurs naturally in estuaries from northern France and Europe and is cultivated in southwestern France for aromatic and pharmacological interests.

For the effective conservation of *A. heterocarpa*, genetic markers providing resolution at the population level are essential although, until now, not available. Here, we report the characterization of 16 new polymorphic microsatellite markers for *A. heterocarpa* and *A. sylvestris* and test their cross‐species transferability in *A. razulii* and *A. archangelica*.

## Main text

### Methods

Plant material was collected across natural populations in France or Germany: two for *A. heterocarpa* (N = 78), three for *A. sylvestris* (N = 98) and one for *A. razulii* (N = 50) and *A. archangelica* (N = 3) (Table 4 in Appendix 1). One or two leaflets were collected from each plant and preserved dried in silica gel. DNA was extracted with the Invitek extraction kit (Invitek, Berlin, Germany). Microsatellites markers were developed from sequences obtained from *A. heterocarpa* and *A. sylvestris* after enrichment by both traditional cloning and high throughput sequencing (GenoScreen, Lille, France) of microsatellite-enriched library [3]. Sequences containing microsatellites were identified using the QDD software [4] and primers were designed using the Primer 3 software [5] using default parameters with 56 °C as annealing temperature. A total of 119 primers pairs for these SSR loci were tested for amplification and genotyping of 4 of each *A. heterocarpa* and *A. sylvestris* individuals using primer extended with M13 sequence for fluorescent labeling [6]. All of them were found to amplify in the both species and among them, 16 showing polymorphisms and consistent peak profile were selected in the final genotyping protocol (Table 1).

**Table 1:** Characteristics of 16 primer pairs for microsatellites loci developed in *A. heterocarpa* and *A. sylvestris*

Locus	Primer sequences (5′ → 3′)		Motif	Range (bp)	Label	Multiplex	GenBank
An23	F:	GAAACAAAATCAAATAGTAATCGCA	AC	75–95	PET	1	MZ065562
	R:	AAATGTAATCTGCACGCGGT					
An35	F:	GGTTGCAACTCAGATGCTGG	GT	175–189	FAM	1	MZ065564
	R:	ACCCTGTGCGTATTGCCTAC					
An69	F:	AGCAAGTGAGGCAAGACCAT	AG	144–176	FAM	2	MZ065568
	R:	CAAACTCTCCTTCACCCCAA					
An71	F:	GAGCATCCTCGAAGAGATCAA	AG	216–262	FAM	1,2	MZ065569
	R:	GGGACCACTCAGAATTGGAA					
An72	F:	TTGGATTCAGGAGAGGACCA	GAT	66–114	FAM	2	MZ065570
	R:	CTTCTCCCAACCAGGATCAG					
An74	F:	GCATTGTAGCCTACTGGAGGG	GT	80–134	VIC	2	MZ065571
	R:	GGCAACAGAGTAGACCTTAACCTG					
An89	F:	GCAACCGCAGCTCATTTTAT	GT	88–106	PET	2	MZ065572
	R:	AAACAAAGGACCAGCCGAC					
An91	F:	CTCGCGTTGACAGGGTTTA	GT	129–143	PET	2	MZ065573
	R:	TCGAGTTCTAGTTGGACAGGG					
An93	F:	GGCAGCTAAGTGAAGCAAAA	AC	170–190	VIC	2	MZ065574
	R:	TGCGCATGTTACTAAGGCTG					
S216	F:	TCTCTGAGTATATATTTTTGGGTGTG	CT	167–179	PET	1	MZ065559
	R:	TCGCAAAATACCCTCATCTC					
An11	F:	GGGACATTCAACACAACATCA	AG	96–128	VIC	1	MZ065561
	R:	CTCTTTCTTGCACCCTTCCA					
An39	F:	TTGGCTGCACTTACATTTGC	CT	212–284	VIC	1	MZ065565
	R:	ATGATAAACCCGGTTGCTTG					
An68	F:	TCCAAAATGCACAGATCCAG	AG	129–171	NED	2	MZ065567
	R:	CTCGTCGAGTTCTACGCTCC					
An32	F:	TGGGTTCATCAAGATTCAAGG	AG	118–174	NED	1	MZ065563
	R:	GTGTGGTCACTGCAAGCATC					
An41	F:	GGGAAACTGAATTAACCGAGC	AG	263–324	PET	1	MZ065566
	R:	CCACTTGTGGTCTCTAACATGG					
S37	F:	CAAAAGTGGATACTAGTTGTGTG	CT	192–224	NED	1	MZ065560
	R:	GCTCTACCATTAGCAAAACC					

Label: fluorophore dye labeling at 5′ end of forward primers

F, forward primer sequence; R, reverse primer sequences; bp, base pair

Multiplexed PCR were achieved using Qiagen Microsatellite Type-It master mix (Qiagen, Hilden, Germany) and cycling conditions were: 15 min at 95 °C; 30 cycles of 30 s at 94 °C, 60 s at 56 °C, 45 s at 72 °C; and 60 °C for 30 min. The fluorescent‐labeled PCR products were run on an ABI 3730 DNA Analyzer (Applied Biosystems, Waltham, MA, USA) at the Genome Transcriptome Facility of Bordeaux. Genotype calling was carried out manually using GeneMapper Software (Applied Biosystems, Waltham, MA, USA).

### Results and discussion

Overall, the developed microsatellite loci were highly polymorphic with a number of alleles per locus ranged from 2 to 21 with a mean of 8 alleles per locus in the five studied *A. heterocarpa* and *A. sylvestris* populations (Table 2). At the population level, the observed heterozygosity ranged from 0.54 to 0.60, and the expected heterozygosity ranged from 0.66 to 0.74. Four out of the 16 loci showed significant deviations from Hardy–Weinberg equilibrium (HWE) in more than one population after correcting for multiple testing (*P* < 0.001; Table 2).

**Table 2:** Genetic diversity characteristics of 16 microsatellites loci for *A. heterocarpa* and *A. sylvestris* populations

	*A. heterocarpa—*Rézé (N = 40)				*A. heterocarpa—*Ile-St-Georges (N = 38)				*A. sylvestris—*Aurice (N = 38)				*A. sylvestris—*La Brède (N = 30)				*A. sylvestris—*Le Bonhomme (N = 30)			
Loci	N_A_	H_E_	H_O_	F_IS_	N_A_	H_E_	H_O_	F_IS_	N_A_	H_E_	H_O_	F_IS_	N_A_	H_E_	H_O_	F_IS_	N_A_	H_E_	H_O_	F_IS_
An23	6	0.75	0.56	0.25	10	0.82	0.68	0.17	5	0.75	0.76	− 0.02	7	0.70	0.50	0.29	4	0.58	0.13	0.79^a^
An35	5	0.67	0.58	0.14	4	0.53	0.55	− 0.05	5	0.54	0.32	0.42	6	0.65	0.70	− 0.08	4	0.56	0.57	− 0.01
An69	3	0.33	0.30	0.09	9	0.83	0.79	0.05	15	0.88	0.76	0.13	5	0.46	0.33	0.29	6	0.47	0.40	0.15
An71	9	0.76	0.32	0.58^a^	18	0.91	0.58	0.36^a^	14	0.88	0.31	0.65^a^	13	0.89	0.72	0.19	15	0.88	0.59	0.34^a^
An72	8	0.80	0.69	0.13	8	0.77	0.76	0.01	11	0.79	0.85	− 0.07	8	0.85	0.83	0.02	13	0.86	0.87	− 0.01
An74	2	0.43	0.10	0.77	4	0.45	0.23	0.49^a^	4	0.55	0.18	0.67^a^	4	0.23	0.18	0.24	2	0.04	0.04	0.00
An89	6	0.74	0.71	0.05	5	0.53	0.27	0.49^a^	8	0.82	0.33	0.60^a^	6	0.71	0.44	0.39	6	0.65	0.53	0.18
An91	6	0.59	0.53	0.12	6	0.51	0.45	0.12	6	0.61	0.58	0.05	6	0.62	0.73	− 0.18	6	0.74	0.69	0.07
An93	5	0.54	0.29	0.47	7	0.69	0.32	0.54^a^	7	0.77	0.21	0.74^a^	7	0.79	0.48	0.40^a^	10	0.89	0.52	0.42^a^
S216	4	0.58	0.58	0.01	4	0.55	0.50	0.10	4	0.64	0.61	0.05	5	0.70	0.43	0.39	3	0.50	0.53	− 0.07
An11	8	0.77	0.75	0.03	9	0.87	0.81	0.07	12	0.84	0.75	0.11	11	0.81	0.67	0.18	8	0.79	0.72	0.09
An39	12	0.82	0.88	− 0.07	21	0.92	0.92	0.00	11	0.76	0.69	0.08	12	0.90	0.76	0.16	7	0.78	0.71	0.09
An68	8	0.77	0.72	0.07	11	0.83	0.79	0.05	15	0.83	0.86	− 0.03	8	0.77	0.83	− 0.09	8	0.74	0.66	0.11
An32	9	0.68	0.74	− 0.09	4	0.29	0.19	0.34	8	0.76	0.50	0.34^a^	6	0.65	0.69	− 0.06	8	0.82	0.60	0.27
An41	12	0.89	0.86	0.04	19	0.91	0.66	0.28^a^	–	–	–	–	10	0.86	0.88	− 0.03	–	–	–	–
S37	8	0.84	0.57	0.33	8	0.77	0.24	0.69^a^	–	–	–	–	6	0.74	0.48	0.36	–	–	–	–
Mean	6.9	0.69	0.57	0.18	9.2	0.70	0.55	0.23	8.9	0.74	0.55	0.27	7.5	0.71	0.60	0.15	7.1	0.66	0.54	0.17

N, number of samples; N_A_, number of alleles (N_A_); H_O_, observed heterozygosity; H_E_, expected heterozygosity; F_IS_, inbreeding coefficient; –, unavailable data

^a^Significant deficit in heterozygotes (P < 0.01)

The cross‐species transferability and polymorphism of the developed SSR markers were further tested in the two other congeneric species (Table 3). In *A. razulii*, only 7 out of the 16 loci amplified consistently and were polymorphic. On the 12 markers tested in *A. archangelica*, 7 amplified successfully and were polymorphic. Note that additional markers amplified inconsistently, suggesting presences of null alleles or limited transferability (3 markers for *A. razulii* and 4 for *A. archangelica*, Table 3).

**Table 3:** Number of alleles and allele range of microsatellite loci in *A. razulii* and *A. archangelica*

Loci	*A. razulii* (N = 50)		*A. archangelica* (N = 3)	
	N_A_	Range (bp)	N_A_	Range (bp)
An23	0	0	1	79
An35	3	177–185	3	169–179
An69	7	140–152	2	142–150
An71	5	212–220	2	216–230 (2)
An72	8	63–96	2	69–72
An74	0	0	1	82 (1)
An89	5	92–102 (20)	1	92 (1)
An91	9	125–143	2	133–135 (1)
An93	0	0	2	170–178
S216	0	0	2	169–179
An11	0	0	2	132–138
An39	2	220–224	3	188–232
An68	0	0	–	–
An32	12	124–156	–	–
An41	2	267–271 (12)	–	–
S37	3	151–171 (3)	–	–

Values in () indicates the number of individuals with at least one PCR amplified signal

N, number of samples; N_A_, number of alleles; bp, base pairs; –, unavailable data

These new polymorphic markers will allow investigating population genetic structure, reproductive system, and potential hybridization within the *Angelica* species complex. Moreover, as numerous *Angelica* species are traditional medicine herbs all over the Eurasia continent, including *A. sylvestris* and *A. archangelica* [7], these microsatellite markers complement the molecular markers previously developed for other congeneric species of particular pharmacological interest [8–10].

## Limitations

- Deviation from HWE: Loci showing marked deficits in heterozygotes in most populations are likely affected by null alleles. However, this should be further investigate with larger sample size because the restricted sampled size of the present study hampered additional statistical test. In addition, more biological insight to support the fact that the species is expect to follow HWE is needed because population substructure or hybridization are likely affecting *Angelica* population and would resulted in departure from HWE.
- Sample size limitation for *A. archangelica*: only 3 individuals have been included to test for transferability and polymorphism for this species. Amplification success and polymorphism should be assessed in a larger sample size to confirm monomorphic loci.

## Data Availability

The genotypic dataset supporting the conclusions of this article available in the Data INRAE repository under DOI, https://doi.org/10.15454/LFMP64. Sequences are accessible in Genbank under accession number MZ065559-MZ065574.

## Appendix 1

See Table 4

**Table 4:** Taxonomic, geographic information (municipality, department number, country) and GPS central coordinates of *Angelica* sp. populations sampling (n = number of individuals) represented in this study

Species	Location, country	*n*	Geographic coordinates	Population name
*A. heterocarpa*	Isle-Saint-Georges (33). FR	38	N44°43′30″-W0°27′28″	Isle St George
*A. heterocarpa*	Rézé (44). FR	40	N47°11′28″-W1°36′6″	Rézé
*A. sylvestris*	La Brède (33). FR	30	N44°40′21″-W0°33′17″	La Brède
*A. sylvestris*	Aurice (40). FR	38	N43°48′38″-W0°35′36″	Aurice
*A. sylvestris*	Le Bonhomme (68). FR	30	N48°8′10″-E7°5′19″	Le Bonhomme
*A. razulii*	Bagnères de Bigorre (64). FR	50	N42°59′48″-E0°7′29″	Bagnères
*A. archangelica*	Hambourg. DE	3	N53°34′18″-E10°00′09″	Hambourg

FR, France; DE, Germany

## Abbreviations

DNA: Deoxyribonucleic acid
HWE: Hardy–Weinberg equilibrium
PCR: Polymerase chain reaction
SSR: Simple sequence repeat
